# Clinical-functional considerations in the association of rheumatoid polyarthritis with chronic hepatitis C

**Published:** 2012

**Authors:** A Clipa, M Şuţa, E Dumitru, L Muflic

**Affiliations:** * RMFB Clinical Hospital in Eforie Nord, Balneology Department, Faculty of Medicine, “Ovidius” University, Constanta; ** Rheumatology Department, IInd Medical Clinic, Clinical Emergency City Hospital in Constanta, Faculty of Medicine, “Ovidius” University, Constanta; *** Gastroenterology Department, Ist Medical Clinic, Clinical Emergency City Hospital in Constanta, Faculty of Medicine, “Ovidius” University, Constanta; **** Clinical Emergency City Hospital in Constanta

## Introduction

Rheumatoid polyarthritis represents the inflammatory rheumatism most frequently met, with a prevalence of approximately 0,5-1% in the general population [**1-3**]. The medium annual incidence in USA is of 70 cases in 100.000 citizens. Although rheumatoid polyarthritis can appear at any age, the most frequently affected are the patients with ages between 30 and 60 years old. Most of the patients present a fluctuant chronic evolution of the disease, which, if not treated in time leads to a progressive irreversible joint destruction, with permanent joint deformities, accompanied by a functional deficit and a considerable reduction of the life expectancy. The management of the patients with rheumatoid polyarthritis can be difficult, and the final purpose of the treatment is the induction of the complete remission. The evaluation of the answer to the treatment is translated, in daily practice, through criteria of remission formulated by ACR (American College of Rheumatology) and EULAR (European League against Rheumatism). The ACR criteria of remission follow six variables such as fatigue, joint pain, the amount of painful joints, the amount of swollen joints, the morning stiffness and the erythrocyte sedimentation rate (ESR) [**4**]. The EULAR criteria of answer to therapy use the DAS28 parameter [**5**].

Currently, viral hepatitis C represents 14-47% of the total amount of acute viral hepatitis recorded in USA and almost 90% of post-transfusion hepatitis. The most significant consequence of the viral hepatitis C, on a long term, is chronic hepatitis (up to 60%), out of which, 10-15% evolves to cirrhosis [**6**]. In the case of post-transfusion chronic hepatitis C, the medium period of time until the appearance of cirrhosis is of 21 years (+/- 10 years), and of the hepatocarcinoma is of 28 years (+/- 11 years) [**7**]. Regarding the extrahepatic manifestations of the viral hepatitis C, the arthropathy is met in a percent of 20%. Unlike the arthritis in the rheumatoid polyarthritis, this is a relatively benign form and it does not associate with bone erosions and joint deformities [**8**]. Chronic hepatitis C is frequently met in patients with rheumatism diseases, 0,65%-5,4% of the patients with rheumatoid polyarthritis being infected with the hepatic virus C [**9**].

## Material and Method

We have conducted a prospective longitudinal study, on a period of five years and a half, from January 2005 to July 2010, which comprised a group of 51 patients with rheumatoid polyarthritis (diagnosed according to the ACR criteria, which were revised in 1987). The patients were diagnosed with associated chronic hepatitis C, and were consecutively admitted in the Medical Clinic III of the County Emergency Hospital in Constanta. There was also a control group, which also contained 51 patients diagnosed with rheumatoid polyarthritis, but without a viral affection.

Initially, the control group contained 56 patients with rheumatoid polyarthritis who concomitantly presented an infection with the hepatic virus C. Afterwards, 5 patients were excluded, because, in the next determinations, they did not have a viral load anymore.

All the patients were evaluated anamnestically, clinically and biologically. The data retained included demographic aspects, the disease duration, the period of time since the first diagnosis, the immunological parameters, the degree of activity of the disease expressed by the DAS28 composite parameter, the morning stiffness, the HAQ (Health Appreciation Questionnaire).

The following clinical variables of activity of the disease were analyzed in the patients with rheumatoid polyarthritis and chronic hepatitis C, but also for in patients in the control group (with a PR without hepatic affection):

• The amount of painful joints
• The amount of swollen joints
• Morning stiffness
• The Visual Analogue Scale (VAS) for the global evaluation of the activity of the disease by the patient
• HAQ
• ESR
• PCR

### Results and Discussions

**Fig. 1:**
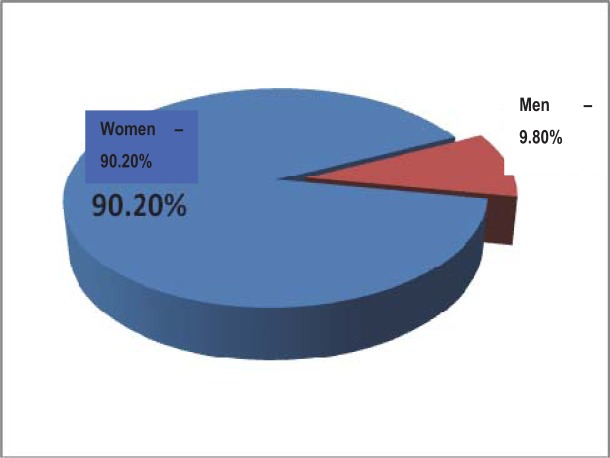
The distribution according to gender

**Fig. 2:**
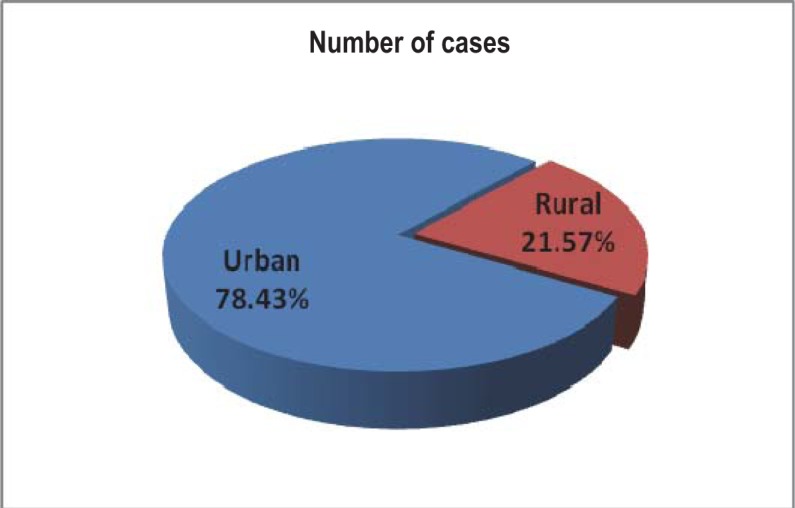
The distribution according to the areas (urban/rural)

From the patients in the studied lot, 46 (90,20%) were women and 5 (9,80%) were men. The obvious preponderance of female patients could be noticed in the study group. The graph below highlights the distribution according to gender of the studied group (**Fig. 1**):

In the studied group of patients, 40 (78,43%) patients came from the urban area and the rest of 11 patients (21,57%) from the rural area. The graph below shows the distribution according to the area (urban/rural) (**Fig. 2**):

The studied patients were aged 43-80 years old. The average age of the group was of 65,16 years, with a medium age of 65 years and a standard deviation of 9,28. For the 51 patients in the control group, the medium age was of 61,37 years (34-86 years) with a standard deviation of 11,55.

**Fig. 3:**
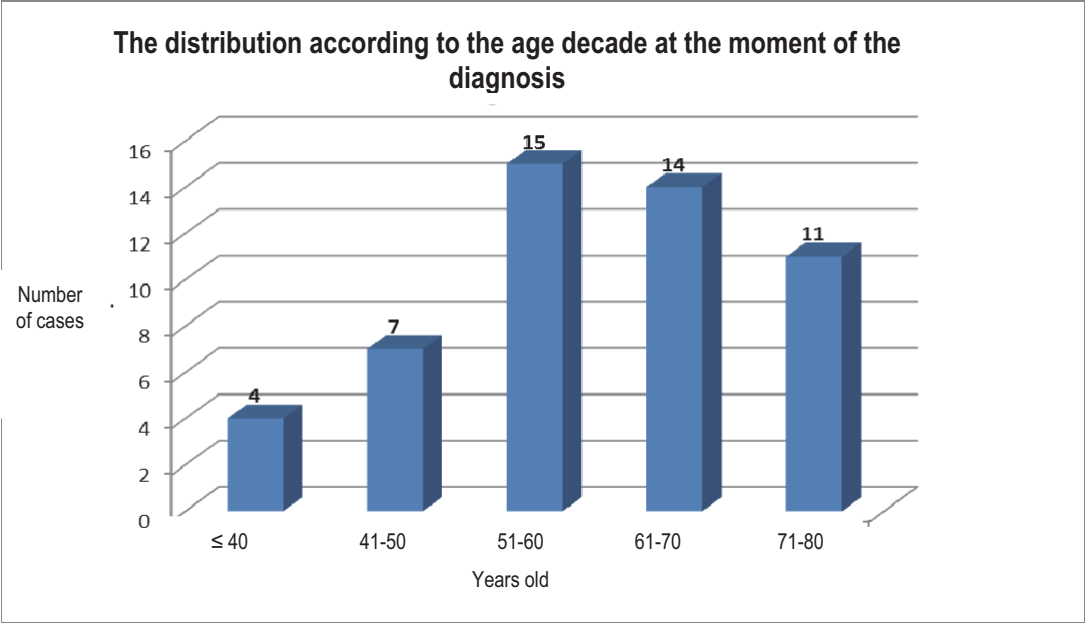
The distribution according to the age decade at the moment of the diagnosis

The distribution of the patients in the study lot on age groups at the moment of the diagnosis, shows a maximum of frequency at the level of the fifth and sixth decades (15, respectively 14 patients) (**Fig. 3**). 11, respectively 7 patients correspond to the seventh and the fourth decades.

**Fig. 4:**
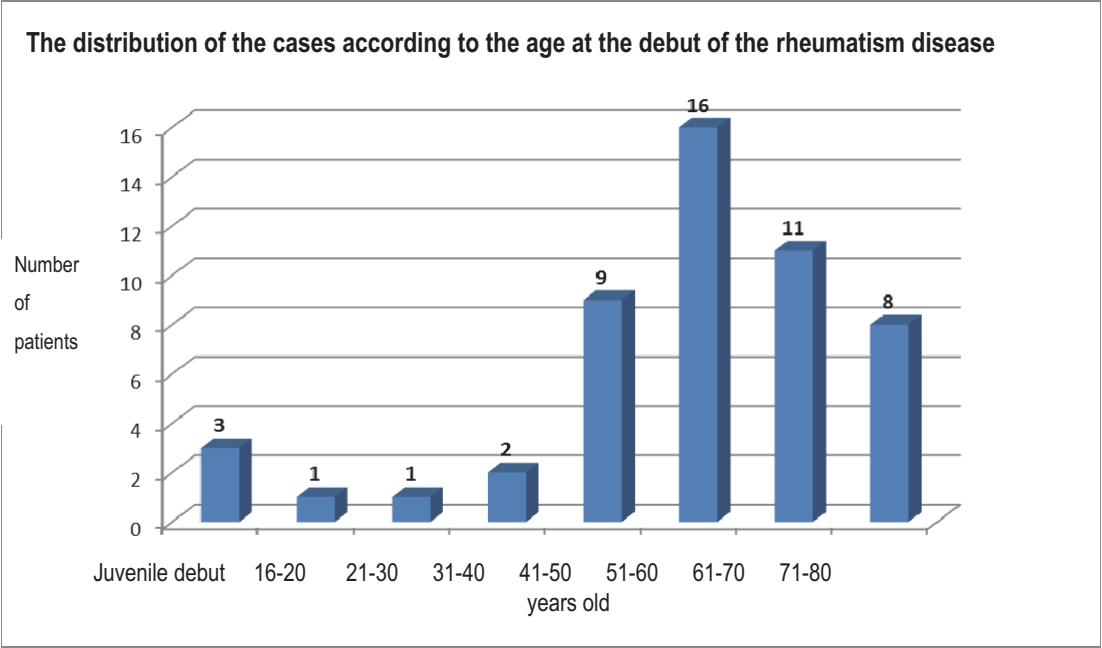
The distribution of the cases according to the age at the debut of the rheumatism disease

The distribution of the patients with rheumatoid polyarthritis and chronic hepatitis C after the debut age of the rheumatism disease shows that 3 patients in the studied group experienced a juvenile debut, and most of them had a debut in the fifth, sixth and fourth decades of life (16, 11 respectively 9 patients) (**Fig. 4**).

Out of the three patients with a juvenile debut in our group, two are females and one is male.

While evaluating the prediagnostic period in the studied lot, we have found out that 27 patients were diagnosed in the precocious or very precocious phase of the rheumatoid polyarthritis (in the first year since the debut of the affection).

Although apparently, the number of the patients diagnosed very late is high [**7**], what should be taken into account is the fact that three of them have had a juvenile debut. In the case of these patients, the lateness of the diagnosis was due to the fact that after the debut, various periods of clinical remission have followed. The diagnosis of rheumatoid polyarthritis could be sustained only when a new episode of arthritis, which has determined the reevaluation of the patients, appeared.

**Fig. 5:**
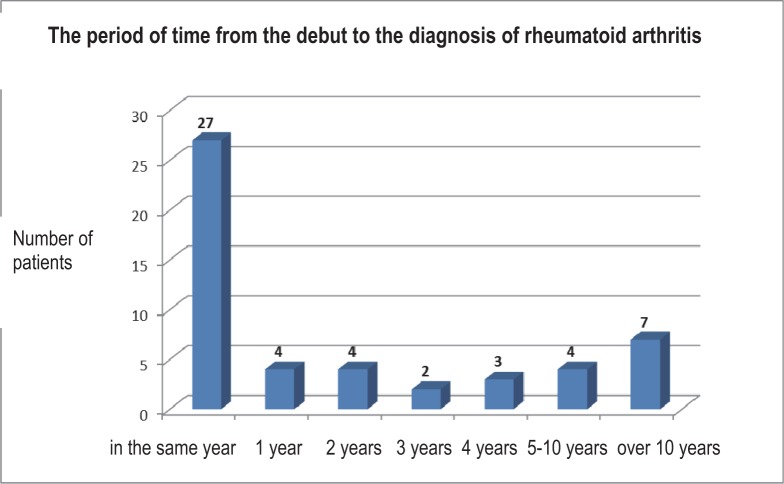
The period of time from the debut to the diagnosis of rheumatoid arthritis

The rest of the patients presented a lateness in the diagnosis of two [**4**], three [**2**], four [**3**] or over five years [**4**] (**Fig. 5**).

**Fig. 6:**
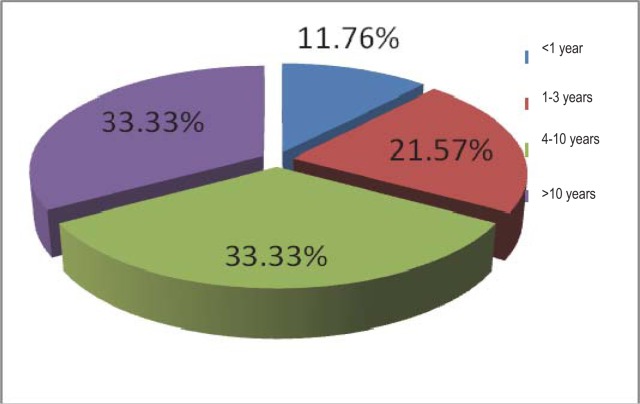
The period of the rheumatoid polyarthritis

The parameter “duration of disease” refers to the time from the debut of the rheumatism symptoms to the moment the patients were included in this study. The evolution of the disease is included in most of the cases 33,33% (n = 17 patients) between 4 and 10 years, following in a descending order the patients with a evolution of the disease between 1 and 3 years - 21,57% (n = 11 patients) and finally, the category with less patients – and a small period of the disease (between 1 and 12 months) - 11,76% (n = 6 patients) (**Fig. 6**).

**Fig. 7:**
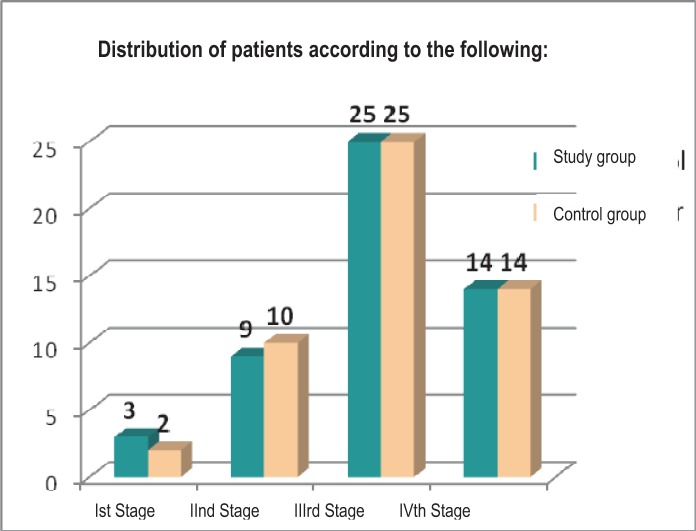
Categories of patients according to the PR stage

As it can be noticed in **Fig. 7**, most of the patients included in the study were diagnosed with stage III rheumatoid polyarthritis. In addition, 5,88% (n=3) are in stage I of the disease, 17,64% of the patients (n=9) are in stage II of the disease, 49,01% (n=25) are in stage III of the disease and 27,45% of the patients (n=14) are in the final stage of the disease.

From the 51 patients studied, 37 (72,5%) presented a positive rheumatoid factor and the rest of 14 (27,5%) presented a negative rheumatoid factor. In the control group – the patients with rheumatoid polyarthritis but without a hepatic affection – 31 (68,9%) presented a positive rheumatoid factor.

We have analyzed the amount of painful joints at the moment the patients were included in the study (different from the moment of the diagnosis).

**Fig. 8:**
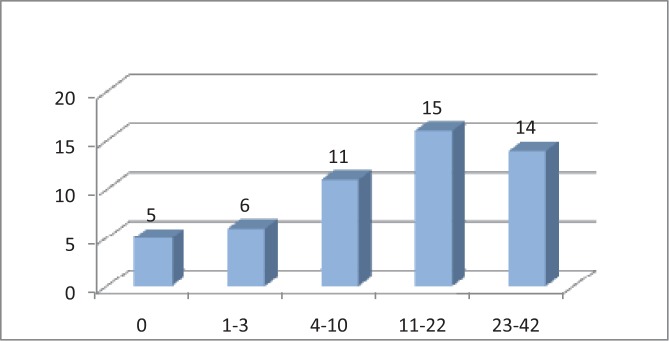
The distribution of the patients according to the amount of painful joints

What is interesting is the distribution of the amount of painful joints in the studied group. Accordingly, most of the patients 29,41% declared 11-12 painful joints, a high number of patients 27,45% declared over 23 painful joints, followed in a descending order by the category 4-10 and 1-3 painful joints, with a percent of 21,57% patients, respectively 11,76% (**Fig. 8**).

**Fig. 9:**
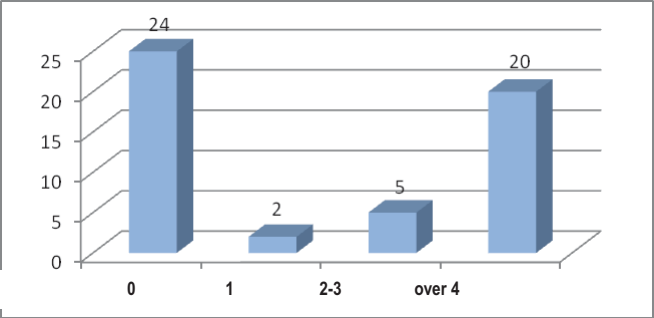
The distribution of the patients according to the amount of swollen joints

As far as the amount of swollen joints at the moment the patients were included in the study (different from the moment of the diagnosis), for the patients with rheumatoid polyarthritis and chronic hepatitis C there is a discrepancy between the declared amount of the painful joints and the amount of the swollen joints observed in the clinical examination. Most of the patients 47,06% did not have any swollen joint (**Fig. 9**).

It can be noticed that the distribution of the values of the amount of painful joints is not the same in the two groups of patients, while there is a similarity as far as the amount of the swollen joints is concerned for the patients of both lots (**Table 1**).

The correlation can suggest the existence of a painful subjective component in the studied group, compared to the control group, possibly explained by the presence of the joint affection due to viral hepatitis C.

**Table 1.** Medium values of NAD/NAT in patients with or without HCV

	**Studied group** **(medium)**	**Control group** **(medium)**	**p**
NAD	15,39	8,98	0,008
NAT	3,06	2,82	0,694

No correlation can be established for the groups of patients in VAS case, the distribution being almost the same (**Table 2**).

**Table 2.** VAS medium value in patients with or without HCV

	**Studied lot** **(medium)**	**Control group** **(medium)**	**p**
VAS	6,47	6,16	0,528

Analyzing the relationship between the visual analogue scale of pain and the other clinical characteristics of the patients with rheumatoid polyarthritis in the studied groups, with the inflammatory biological syndrome, we have noticed significant associations between it and the amount of painful joints, swollen joints, morning stiffness, DAS28, ESR, but there were no correlations between ESR and HAQ, PCR (**Table 3**).

**Table 3.** The relationship between VAS and the other parameters – studied group

	**ESR**
NAD	p=0,001
NAT	p=0,019
Morning stiffness	p=0,004
HAQ	p=0,051
DAS28	p=0,000
ESR	p=0,006
PCR	p=0,147

We have also obtained similar results for the patients in the control group. We could not notice any correlation between VAS and the morning stiffness, ESR, PCR (**Table 4**).

**Table 4.** The relationship between VAS and the other parameters – control group

	**VAS**
NAD	p=0,002
NAT	p=0,000
Morning stiffness	p=0,089
HAQ	p=0,000
DAS28	p=0,000
ESR	p=0,082
PCR	p=0,266

The patients were asked to answer to a questionnaire, which was conceived so that it could evaluate the difficulty of doing daily current exercises such as the following: grabbing objects, getting dressed, eating, assuring the personal hygiene, climbing up and down the means of conveyance. The health evaluation questionnaire (HAQ) was made up of 36 questions, which covered 8 areas of activity. The questionnaire offered a score with limits between 0 (without difficulty) and 3 (impossible). It was calculated by taking into account the highest score of each category and the resulted sum was divided to the number of categories. High values of HAQ represented a high degree of divisibility in the execution of the daily current activities.

No correlation could be established between the two groups for HAQ, similar to VAS, by determining p=0,579 (**Table 5**).

**Table 5.** Medium value of HAQ in patients with or without HCV

	**Study group** **(medium)**	**Control group** **(medium)**	**p**
HAQ	2,20	2,07	0,579

**Fig. 10:**
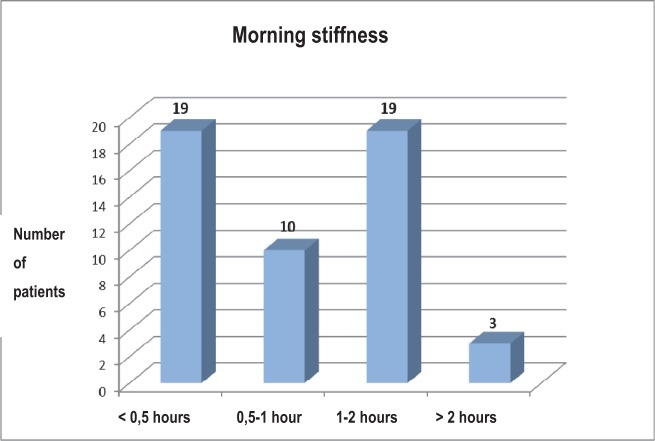
The distribution of the patients according to the period of the morning stiffness

The distribution of the patients in the study group, from the point of view of the morning stiffness, showed that most of them presented a period of less than 30 minutes and between one and two hours, and, fewer patients (n=3) declared a period of over two hours of the morning stiffness (**Fig. 10**).

We have analyzed the relationship between the period of the morning stiffness and the other clinical parameters, both in the study group and the control group. It is important to mention the significant association, from a statistical point of view, of the period of the morning stiffness and the amount of painful, swollen joints, VAS, DAS28, VSH for the patients with rheumatoid arthritis and chronic hepatitis C (**Table 6**).

**Table 6.** Relationship between the morning stiffness and the other elements – study group

	**Morning stiffness**
NAD	p=0,000
NAT	p=0,047
VAS	p=0,004
HAQ	p=0,058
DAS28	p=0,001
VSH	p=0,003
PCR	p=0,067

In the control group, the period of the morning stiffness did not correlate with VAS, HAQ, respectively PCR (**Table 7**).

**Table 7.** Relationship between the morning stiffness and the other elements – control group

	**Morning stiffness**
NAD	p=0,001
NAT	p=0,001
VAS	p=0,089
HAQ	p=0,810
DAS28	p=0,000
VSH	p=0,047
PCR	p=0,383

We compared the clinical parameters, the DAS28 composite parameter and HAQ functional parameter, according to the age groups in the two groups of patients (control and study).

We could not identify significant differences from a statistical point of view between the morning stiffness and the distribution according to age groups, or, between the latter and HAQ. A similar situation was also met in the case of the patients in the control group. However, we have noticed a medium index of activity of the disease and the medium value of VAS, which were higher in patients over 60 years old (p=0,017, respectively p=0,028) in the studied group (**Table 8**), compared to the control group which did not record this type of correlations (**Table 9**).

**Table 8.** Characteristics of the patients with PR and HCV according to their age

	**40–59 years old** **(*n *= 16)**	**⩾60 years old** **(*n *= 35)**	**p**
VAS	5,93 ± 2,78	6,66 ± 2,77	0,028
DAS28	5,04 ± 1,34	5,64 ± 1,68	0,017
Morning stiffness	52,69 ± 65,27	49,31 ± 54,02	0,154
HAQ	2,15 ± 0,78	2,23± 0,76	0,559

**Table 9.** Characteristics of the patients with PR according to their age

	**30–59 years old** **(*n *= 23)**	**⩾60 years old** **(*n *= 28)**	**p**
VAS	6,26 ± 2,61	6,07 ± 2,70	0,932
DAS28	4,93 ± 1,47	4,75 ± 1,72	0,614
Morning stiffness	43,04 ± 45,47	33,93 ± 41,04	0,818
HAQ	2,18 ± 0,67	1,98± 0,92	0,593
